# *In-vivo *generation of bone via endochondral ossification by *in-vitro *chondrogenic priming of adult human and rat mesenchymal stem cells

**DOI:** 10.1186/1471-2474-12-31

**Published:** 2011-01-31

**Authors:** Eric Farrell, Sanne K Both, Kathrin I Odörfer, Wendy Koevoet, Nicole Kops, Fergal J O'Brien, Robert J Baatenburg de Jong, Jan A Verhaar, Vincent Cuijpers, John Jansen, Reinhold G Erben, Gerjo JVM van Osch

**Affiliations:** 1 Department of Orthopaedics, Erasmus MC University Medical Centre Rotterdam, the Netherlands; 2 Department of Otorhinolaryngology, Erasmus MC University Medical Centre Rotterdam, the Netherlands; 3 Department of Biomaterials, Radboud University Medical Center Nijmegen; 4 Department of Biomedical Sciences, University of Veterinary Medicine, Vienna, Austria; 5 Department of Anatomy, Royal College of Surgeons in Ireland, Dublin, Ireland; 6 Trinity Centre for Bioengineering, Trinity College Dublin, Ireland

## Abstract

**Background:**

Bone grafts are required to repair large bone defects after tumour resection or large trauma. The availability of patients' own bone tissue that can be used for these procedures is limited. Thus far bone tissue engineering has not lead to an implant which could be used as alternative in bone replacement surgery. This is mainly due to problems of vascularisation of the implanted tissues leading to core necrosis and implant failure. Recently it was discovered that embryonic stem cells can form bone via the endochondral pathway, thereby turning in-vitro created cartilage into bone in-vivo. In this study we investigated the potential of human adult mesenchymal stem cells to form bone via the endochondral pathway.

**Methods:**

MSCs were cultured for 28 days in chondrogenic, osteogenic or control medium prior to implantation. To further optimise this process we induced mineralisation in the chondrogenic constructs before implantation by changing to osteogenic medium during the last 7 days of culture.

**Results:**

After 8 weeks of subcutaneous implantation in mice, bone and bone marrow formation was observed in 8 of 9 constructs cultured in chondrogenic medium. No bone was observed in any samples cultured in osteogenic medium. Switch to osteogenic medium for 7 days prevented formation of bone in-vivo. Addition of β-glycerophosphate to chondrogenic medium during the last 7 days in culture induced mineralisation of the matrix and still enabled formation of bone and marrow in both human and rat MSC cultures. To determine whether bone was formed by the host or by the implanted tissue we used an immunocompetent transgenic rat model. Thereby we found that osteoblasts in the bone were almost entirely of host origin but the osteocytes are of both host and donor origin.

**Conclusions:**

The preliminary data presented in this manuscript demonstrates that chondrogenic priming of MSCs leads to bone formation *in vivo *using both human and rat cells. Furthermore, addition of β-glycerophosphate to the chondrogenic medium did not hamper this process. Using transgenic animals we also demonstrated that both host and donor cells played a role in bone formation. In conclusion these data indicate that in-vitro chondrogenic differentiation of human MSCs could lead to an alternative and superior approach for bone tissue engineering.

## Introduction

Bone can be damaged by trauma or disease and often bone graft substitutes are then needed for repair. Substitute bone can be derived from the patient (autograft) or from a donor (allograft). The common treatment is to use autologous bone grafts but this method has its drawbacks. It causes the generation of a second surgical site with increased donor site morbidity. Secondly, availability of autologous bone is limited [1]. With the other option, using allograft material, there are risks of immune reaction and disease transmission [2]. For this reason, there is a huge interest in developing new strategies for bone replacement. Marrow derived progenitor cells of adults represent a promising source of therapeutic tool and are known to differentiate along various mesenchymal lineages. The use of adult bone marrow stromal cells (MSCs) to achieve bone and cartilage formation and repair have met with less success and more problems than expected [1]. In relation to bone formation, one of the largest problems has been nutrient delivery and waste removal associated with a lack of vasculature in implanted tissues leading to core necrosis and implant failure. It is clear that vascularisation is a critical consideration for any regenerative medicine approach [3,4]. Cartilage is an avascular tissue and, hence, does not suffer from this problem. However, regenerative medicine approaches to cartilage regeneration have also met with problems [5], mainly because of the tendency of MSCs to naturally progress from forming stable, collagen type II expressing, cartilage to a more hypertophic phenotype characterised by expression of collagen type X.

In a recent paper we hypothesised that the natural tendency of chondrogenically primed MSCs to become hypertrophic might be a very desirable trait for bone tissue engineering applications [6]. MSCs have been shown to progress along similar stages of endochondral ossification as observed during development [7]. Recent successes in the induction of endochondral ossification from embryonic stem cells and murine bone marrow cells supported the feasibility of such an approach [8-10]. There are several rationales behind the hypothesis that this route of bone formation would be more successful than intramembranous ossification. Firstly, chondrocytes normally reside in an avascular tissue and as a result are "designed" to function in a low oxygen environment, similar to what they would encounter upon implantation into an unvascularised region [11]. Secondly, as stated, MSCs under *in-vitro *conditions (almost) always become hypertrophic when cultured chondrogenically, the next step in the endochondral ossification pathway [7,12]. Thirdly, the release of factors from primed chondrogenic cells progressing along the endochondral route would be much more complex and controlled spatiotemporally than any growth factor combination we could devise in order to improve *in-vivo *vascularisation and bone formation. Previously [6], we demonstrated that chondrogenically primed MSC seeded scaffolds did indeed survive 4 weeks *in-vivo *without core necrosis as evaluated histologically. Furthermore, we observed blood vessels in the chondrogenic samples only and data suggested that this was due to release of VEGF from these constructs as measured *in-vitro *in chondrogenic pellets. However, we did not observe bone formation in any of the samples *in-vivo *after 4 weeks. We hypothesised that either samples were not primed for long enough *in-vitro *or they were not maintained *in-vivo *for long enough to allow the process to occur.

In the current experiment we cultured MSC seeded scaffolds for a longer period *in-vitro *to allow cells to migrate into the scaffolds prior to priming and to potentially form more matrix prior to implantation. In addition, samples were maintained *in-vivo *for a minimum of 8 weeks. Our aim was to answer 3 specific questions. Firstly can adult human MSC seeded scaffolds undergo endochondral ossification *in-vivo *to form bone for the purposes of bone repair/replacement? Secondly, can this process be further optimised by allowing mineralisation to occur *in-vitro *for a brief period of time before implantation, thereby speeding up or enhancing the quantity of bone formed. Thirdly, what is the role of the donor and host cells in the process of endochondral ossification? To answer this final question we used a transgenic rat model ubiquitously expressing human placental alkaline phosphatase (hPLAP) as a recipient of wild type cells [13,14].

## Materials and methods

### human bone marrow cell culture

Bone marrow aspirates were obtained from three donors, 47, 57 and 69 years of age undergoing total hip arthroplasty after informed consent with approval of the local medical ethical committee (METC2004-142). The aspirates were plated as previously described [6].

To create a pellet, suspensions of 200,000 cells per 15 ml tube were centrifuged at 200 g for 8 minutes. For the scaffolds, suspensions of detached cells were seeded with 1*10^6 ^cells per scaffold, divided into 500,000 cells in 100 μl on each side of the Collagen-GAG scaffolds as described previously [15]. The constructs were cultured for 7 days in medium as used for expansion (control medium). Afterwards all samples were either maintained in control medium or replaced with chondrogenic or osteogenic medium for 28 days. Half of the medium was replaced every 3 days.

Chondrogenic medium consisted of high-glucose DMEM containing 50 mg/mL of gentamicin and 1.5 mg/mL of Fungizone (Invitrogen) 25 μg/ml L-ascorbic acid 2-phosphate, 100 mM of sodium pyruvate (Invitrogen), 1:100 insulin-transferrin-selenium (ITS; BD Biosciences, Bedford, MA), 10 ng/mL of transforming growth factor beta-2 (TGF-b2), (R&D Systems, Abingdon, United Kingdom) and 100 nM dexamethasone (Sigma, St. Louis, MO). The osteogenic medium consisted DMEM containing 10% fetal calf serum (Gibco, selected batch), gentamicin and 1.5 mg/mL of Fungizone (Invitrogen) 0.1 mM L-ascorbic acid 2-phosphate, 10 mM beta-glycerol phosphate, 100 nM dexamethasone.

To investigate if bone formation *in vivo *can be enhanced by allowing mineralisation to occur before implantation, we have applied chondrogenic medium for 21 days and then switched to mineralizing medium conditions for the last 7 days of culture. For the **switch 1 **condition the chondrogenic medium was replaced after 21 days of culture with osteogenic medium for the remaining period of 7 days. For the **switch 2 **condition after 21 days of culture in chondrogenic medium, 10 mM beta-glycerol phosphate (as a source of phosphate to allow for mineralization) was added to the chondrogenic medium for the remaining period of 7 days.

### Gene expression analysis

To confirm chondrogenic potential of MSCs prior to implantation, gene expression analysis of GAPDH, Sox9, cbfa1, collagen type II and collagen type X was performed as described previously [16] In addition, samples cultured as pellets were harvested from each MSC donor, fixed in 4% phosphate buffered formalin and embedded in paraffin for collagen type II immunohistochemistry (II-II6B3 antibody, 1:100; Developmental Studies Hybridoma Bank, Iowa City, IA, under contract N01-HD-6-2915 from the National Institute of Child Health and Human Development).

### In vivo implantation of hMSC

To evaluate bone formation, cultured constructs were implanted subcutaneously in athymic mice (Balb/C nudes, CDL Nijmegen). For each donor, 3 constructs of each condition were implanted. Before surgery, the skin on both lateral sites of the spine was cleaned with 70% alcohol and 4 subcutaneous pockets were created in each mouse. The tissue engineered samples or pellets were inserted and the pockets closed. Three empty scaffolds were also implanted. Two of these were maintained for the duration of the culture period in expansion medium and one of these in chondrogenic medium. Eight and fourteen weeks after surgery, the animals were euthanized by CO_2_. The explanted samples were fixed in 4% paraformaldehyde, decalcified in formic acid and embedded in paraffin. The experiments were approved by the Dutch animal experiment committee.

### Micro CT imaging

All samples were scanned using micro-CT (Skyscan model 1072, Kontich, Belgium) with a source of 50 kV/98mA without using a filter (resolution 8.1 μm per pixel). Each sample was rotated 180 degrees with a rotation step of 0.90 degrees, exposure time 2.9 seconds. 3D reconstruction, analysis and visualizations were made with NRecon version 1.6, CT-analyzer V1.9 (Skyscan) and 3D-Doctor™ (Able Software Corp., Lexington, United States).

### Histomorphometrical analysis

Sections were stained with haematoxylin-eosin and evaluated for presence or absence of bone. A Fisher exact test was used to evaluate statistical significance. Histomorphometry was performed on 2-4 sections of each sample. From each section, low magnification digital images were made, images were pseudo colored and measurements were performed with image analyses techniques (Leica Qwin Pro-image analysis system, Wetzlar, Germany) to obtain the percentage of bone, bone marrow and other tissue.

### Rat MSC isolation, culture and implantation

MSCs from 5 month-old inbred wild-type Fischer 344 (F344) rats were isolated and cultured according to standard procedures as described elsewhere [15]. Culture and scaffold seeding was performed exactly as for the human MSCs as described above. The second switch condition was employed for the rat component of this study. Following 5 weeks *in vitro*, three constructs (scaffolds) of each condition were implanted subcutaneously into immunocompetent co-isogenic hPLAP-transgenic (human Placental Alkaline Phosphatase) F344 rats for 8 weeks. Animals were sacrificed by exsanguination under ketamine/xylazine anesthesia. Scaffolds were harvested and fixed in 40% ethanol at 4°C for 48 h, dehydrated and embedded in modified methylmetacrylate [17].

### Immunohistochemistry for collagen type II

To analyze collagen type II expression, sections were incubated with 0.1% pronase (Sigma, St Louis, MO) for antigen retrieval and 1% hyaluronidase (Sigma, St Louis, MO). Sections were incubated for 2 h at room temperature with mouse monoclonal antibody against collagen type II (II-II6B3 antibody, 1:100; Developmental Studies Hybridoma Bank, Iowa City, IA, under contract N01-HD-6-2915 from the NICHD).

### hPLAP immunohistochemical staining

For histochemical staining of the marker enzyme hPLAP deplastisized sections were rehydrated and heated at 65°C for 30 min in deionized water to block endogenous alkaline phosphatase activity. Cells expressing hPLAP were histochemically stained by incubation with an AP substrate (TRIS-HCl buffer (0.2 M, pH 8.5) containing Naphtol AS-MX 0.3 mg/ml (Sigma) and New Fuchsin 0.1 mg/ml (Chroma)) at room temperature for 1 hour and counterstained with haematoxylin.

## Results

### Bone formation occurs following chondrogenic priming of MSCs

**Figure 1:**
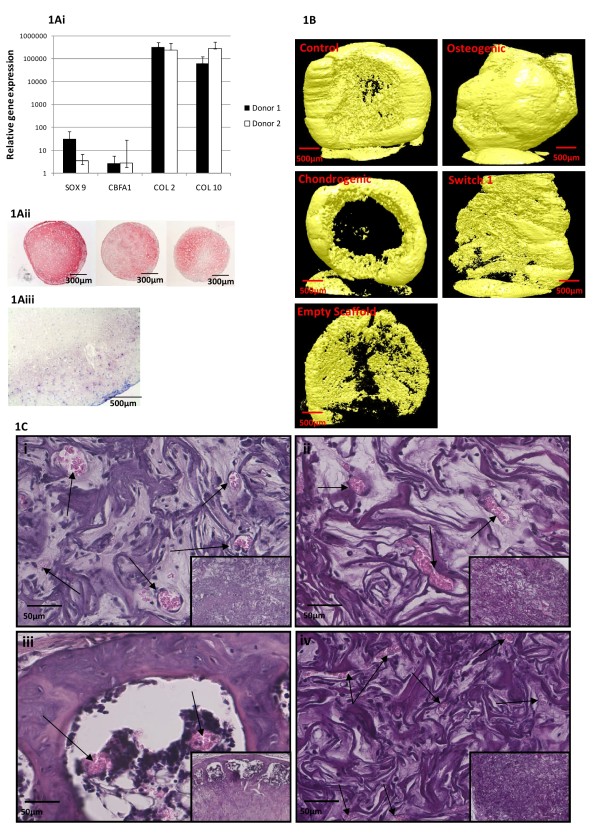
**Chondrogenic priming of MSCs seeded into Collagen GAG scaffolds in-vitro leads to bone formation in-vivo**. Figure 1A; Chondrogenic potential was confirmed in all three donors by PCR (donors 1 and 2, expression relative to undifferentiated donor matched controls) and collagen type II immunohistochemistry (Figure 1Aii Donors 1-3). Figure 1Aiii, Toluidine Blue staining of a chondrogenically primed scaffold prior to implantation. Figure 1B Micro computed tomography of retrieved constructs (resolution 8.1 μm per pixel). The pattern of bone formation observed histologically matched closely with these images showing bone tissue at the edges of the constructs. Mineralised matrix that did not form bone was also observed in all constructs as well as empty scaffold controls. Figure 1C; Hameatoxylin and Eosin staining of bone formation in chondrogenically primed constructs (1Ciii) as compared to constructs cultured in osteogenic (Figure 1Cii) medium for 4 weeks. While osteogenically primed samples were more mineralised compared to *in-vitro *samples, no true bone formation was observed. Switch from chondrogenic to osteogenic medium for 7 days also prevented *in-vivo *bone formation (Figure 1Civ). Insets represent lower magnification images of the constructs. Arrow indicate blood vessels in each construct.

In order to confirm chondrogenic differentiation of MSCs prior to implantation, chondrogenically primed samples were analysed by realtime qRT-PCR for common chondrogenic and hypertrophic markers and compared to monolayer controls. Expression of sox 9, collagen type II, cbfα1 and collagen type X were significantly elevated compared to monolayer control levels (Control values set to 1 for each gene, Figure 1Ai). Immunohistochemistry for collagen type II performed on pellets cultures also confirmed the chondrogenic potential of MSCs from all donors (Figure 1Aii).

Upon culture of MSCs in scaffolds under chondrogenic conditions and subsequent implantation, bone formation was observed in 5 out of 6 scaffolds (Figure 1C iii,) from two different MSC donors. In a third donor bone formation did not occur in scaffolds however was observed in chondrogenically primed pellets, which will be discussed further. Bone formation occurred mainly at the edges of scaffolds and appeared to be progressing inwards by the presence of calcified cartilage interior to the bone and marrow regions. This structural organisation of the bone regions was confirmed by μCT (Figure 1B). Histomorphometry revealed that 11 ± 5% of the construct area was bone tissue, 5% was bone marrow and the rest was qualified as other tissue, consisting of remnants of scaffold material, cartilaginous tissue and fibrous tissue. The newly formed bone tissue was lined with cells resembling osteoblasts and was associated with bone marrow replete with red blood cells, stroma and fat cells. In no other treatment condition was bone or marrow tissue observed. In the scaffolds cultured in control medium (Figure 1C, n = 6, from 2 donors), or osteogenic medium (Figure 1C, n = 6 from 2 donors) or in scaffolds switched to osteogenic medium for 1 week (Figure 1C, n = 6 from 2 donors), mineralised tissue was observed both histologically and by μCT (Figure 1B), but this lacked any significant structural organisation or surrounding marrow. This mineralisation was also observed to a lesser degree within empty scaffolds after 8 weeks *in vivo*.

### Osteogenic culture or switch prevents endochondral ossification but addition of β-glycerophosphate does not

**Figure 2:**
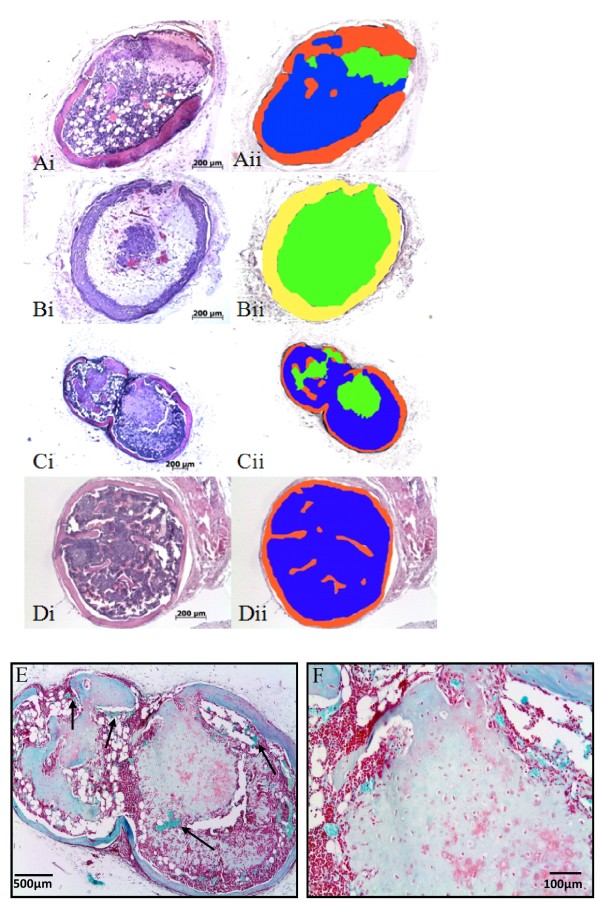
**Osteogenic culture or switch prevents endochondral ossification but addition of β-glycerophosphate does not**. Representative hematoxilin-eosin stained slides of implanted pellets in immune deficient mice for 8 weeks. Primed chondrogenically bone, cartilage and marrow stroma are visible (Ai). For the switch 1 condition the chondrogenic medium was replaced during the last 7 days for osteogenic medium which resulted in cartilage-like tissue in the inside and undefined tissue on the outside (Bi). For the switch 2 condition β-glycerophosphate was added during the last 7 days of culture and bone, cartilage and marrow stroma are observed (Ci). When the chondrogenic primed pellets were implanted for 14 weeks only bone and marrow stroma were visible. For quantitative analysis all pictures were pseudo colored, red (bone), blue (marrow stroma) green (cartilage), undefined tissue (yellow) (Aii, Bii, Cii, Dii). Figure 2, E and F show Safranin O staining of *in vitro *chondrogenically cultured pellets retrieved after 8 week *in vivo*. Weakly positive staining demonstrates the presence of glycosaminoglycans within a cartilage matrix being degraded to make way for bone and marrow formation which surrounds the remnants of the cartilage matrix.

Following the results observed in donors 1 and 2, the osteogenic condition was discontinued with donor 3. In addition to the complete switch to serum containing osteogenic medium for the last culture week, samples were maintained on chondrogenic medium with β-glycerophosphate to achieve mineralisation of the matrix as observed previously [6]. This experiment was performed with cell-seeded scaffolds and pellet cultures. Bone formation was observed in all pellets that were primed chondrogenically (Figure 2Ai) confirming results of the experiments with scaffolds. Once again, under the initial switch conditions of culturing cells for 1 week in osteogenic medium after 3 weeks in chondrogenic medium, no endochondral ossification or bone formation was observed (Figure 2Bi). Only one of the three implanted pellets of the second switch condition where β-glycerophosphate was added to the chondrogenic medium, was retrieved. Interestingly, in this condition bone formation was observed to occur similarly to the chondrogenic condition (Figure 2Ci). Once again, a marrow stroma was also observed within the pellets around the area of bone formation. This effect was also observed in all 3 scaffold samples that were cultured under identical conditions in the rat study. Addition of β-glycerophosphate did not prevent bone formation *in vivo*. Histomorphometry (Figure 2Aii, Bii, Cii) revealed that in the pellet constructs with bone formation 32 ± 10% of the construct area consisted of bone tissue, and 37 ± 24% of bone marrow. The rest of the area (39 ± 27%) was cartilage as confirmed by positive collagen type II immunohistochemistry. This data is presented in Table 1. Safranin O staining of these pellets showed the presence of small amounts of GAGs remaining in the cartilage like matrix. This indicates that in this phase most proteoglycans have been degraded, a process which occurs during endochondral ossification. Figures 2E and 2F demonstrate all stages of endochondral ossification in the same pellet, cartilage degradation, blood vessel invasion and bone and marrow formation.

**Table 1:** Description of treatment conditions and semi-quantitative measurement of bone and marrow formation in scaffold constructs and pellets.

Donor	Treatment	Implanted	Bone formed	% Bone	% Marrow
**1**	Control in scaffold	3	0/3	0	0
	Osteogenic in scaffold	3	0/3	0	0
	Chondrogenic in scaffold	3	3/3	9 ± 3	8 ± 7
	Switch 1 (switch to osteogenic culture) in scaffold	3	0/3	0	0

**2**	Control in scaffold	3	0/3	0	0
	Osteogenic in scaffold	3	0/3	0	0
	Chondrogenic in scaffold	3	2/3	13 ± 7	6 ± 7
	Switch 1 (switch to osteogenic culture) in scaffold	3	0/3	0	0

**3**	Chondrogenic in scaffold	3*			
	Switch 1 (switch to osteogenic culture) in scaffold	3*			
	Switch 2 (+β-glycerophosphate) in scaffold	3*			

**3**	Chondrogenic in pellet	3	3/3	32 ± 10	37 ± 24
	Switch 1 (switch to osteogenic culture) in pellet	3	0/2	0	0
	Switch 2 (+β-glycerophosphate) in pellet	3	1/1	23	52
	14 weeks chondrogenic in pellet	3	2/2	24 ± 7	76 ± 7

Results are average ± SD. *Donor 3 scaffolds were not analysed because of poor cell seeding as determined in pre-implantation samples by histology and RNA content.

We also retrieved a chondrogenically primed pellet implanted *in-vivo *for 14 weeks. This construct had 24% ± 7 bone tissue and 76% ± 7 marrow. No other tissue was present. Bone tissue was located at the outer rim of the construct (Figure 2Di and 2ii). As stated above no bone formation was observed in the scaffolds from donor 3. However, this was due to poor cell seeding of the scaffolds as identified by a very low cell number in scaffolds prior to implantation as identified by histological assessment of un-implanted samples.

### Role of host and donor cells

**Figure 3:**
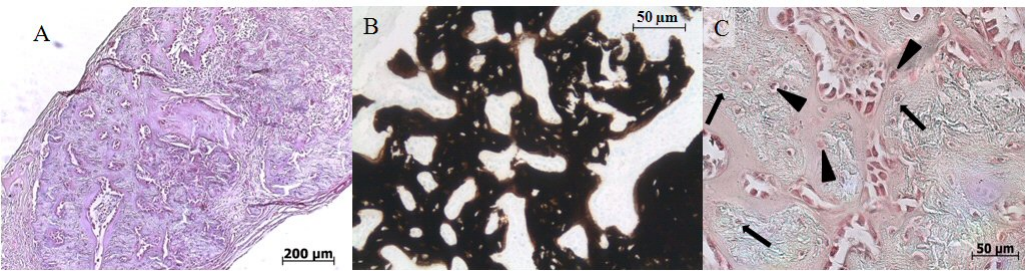
**Role of host and donor cells**. By implanting in transgenic rats we can distinguish between donor and host with a hPLAP immunohistochemical staining. Overview of the implanted scaffold in which bone and bone marrow can be observed on hematoxilin-eosin A) and von Kossa (B) staining. All osteoblasts are stained red indicating they are from the host (C). The osteocytes however embedded in the bone are of both host (arrowheads) and donor origin (arrows).

In order to determine the role of host and donor cells in the generation of tissue engineered bone via endochondral ossification the described experiments were repeated in an immunocompetent model using hPLAP transgenic rats. This model enabled specific staining of alkaline phosphatase activity to distinguish host and donor cells from one another to determine the origin of the various cells observed in the constructs upon retrieval. Similar to the implanted human cells, bone formation occurred in all chondrogenically primed samples (Figure 3A&3B). Once again no bone formation was observed in any of the constructs that were cultured in osteogenic medium (data not shown). As was observed in the switch 2 condition, maintained in chondrogenic medium for 3 weeks and simply supplied with β-glycerophosphate during the last week, bone formation was observed in two of the three constructs (data not shown). Most important in this experiment was the question of whether the bone that was formed was host or donor derived. Staining for the human placental alkaline phosphatase activity found only in host cells demonstrated that the osteoblasts were almost entirely of host origin. Interestingly, there was a mixed population of osteocytes embedded in the bone that stained both positively and negatively(Figure 3C), suggesting that donor-derived cells do indeed participate in bone formation at earlier stages after implantation.

## Discussion

Tissue engineering approaches to bone repair have thus far been disappointing. Recent interest has focused on the process of endochondral ossification as a possible means to generate bone for regenerative medicine purposes [6,8,18-20]. The release profile of factors that occurs during endochondral ossification is complex and coordinates the formation of bone from a cartilage template [21]. Here we show that chondrogenically differentiated adult human and rat MSCs seeded into collagen GAG scaffolds give rise to bone formation via endochondral ossification *in-vivo*. Previously, it was demonstrated that this was possible with murine embryonic stem cells [8] as well as murine adult bone marrow stromal cells [9,10]. The data from our study are also supported by the recent publication by Chan *et al *[22] demonstrating that endochondral ossification is required for haematopoietic stem cell niche formation with a subpopulation of foetal progenitor cells giving rise to bone with a marrow cavity only if they would normally undergo endochondral ossification as opposed to intramembranous ossification. Even more recently, Janicki *et al *[23] demonstrated the same mechanism of bone formation via endochondral ossification using human MSCs and β-tricalcium phosphate with a 6 week *in-vitro *chondrogenic pre-culture.

Initial results presented in this manuscript demonstrate that eight weeks of implantation was not sufficient to ossify the complete construct. In case of a completely cartilaginous construct (such as the pellets we used) the remaining tissue is cartilage and the sample harvested after 14 weeks demonstrated that the complete construct is subsequently turned into bone and bone marrow. The bone is then only localised at the outer rim, probably due to a lack of mechanical stimulation that is prerequisite for bone maintenance. The technical problems associated with homogenous cell seeding will likely become relevant when upscaling the procedure towards application in patients. Use of bioreactors to improve cell seeding efficiency and also mechanical integrity could be considered [24-26].

### Addition of β-glycerophosphate

An important consideration in the generation of bone via endochondral ossification is the optimum differentiation stage at which one can implant. Ideally, the further along the differentiation pathway a construct is prior to implantation the faster it would fulfil its role *in-vivo*. To assess this we cultured scaffolds in both osteogenic medium as a negative control of bone formation and in chondrogenic medium for 3 weeks with a switch to standard osteogenic medium for one week (switch 1) to begin the osteogenic differentiation process. Despite the brief period of exposure to these culture conditions, no bone formation was observed *in-vivo*. We hypothesised this was due to a lack of vascularisation due to reduced release of pro-angiogenic factors that we had previously observed *in-vitro *[6]. However upon close inspection, blood vessels were observed in all 4 culture conditions. As a further evaluation of the effect of the presence of mineralisation before implantation, we simply added β-glycerophosphate to the chondrogenic media for 1 week which we had also shown previously to cause mineralisation. Unfortunately only a single pellet was retrieved in the human MSC pellets as the other two could not be located. However, bone formation under this switch 2 (glycerophosphate only) condition was also observed in 3 out of 3 scaffolds retrieved in the rat study (Data not shown). Thus we are confident that addition of glycerophosphate for a week will not prevent endochondral ossification as we observed in the switch 1 (full osteogenic medium switch). These findings would suggest that pre-mineralisation alone will not prevent the process of endochondral ossification occurring *in-vivo*. It is likely another factor in the osteogenic medium that prevents bone formation *in-vivo*, possibly the presence of serum. The lack of retrieved samples in the human MSC study can not be ignored however as it is possible that these samples could not be retrieved because they were resorbed by the host. Pre-mineralisation might offer the advantage of stiffer scaffolds upon implantation which would greatly improve the options in load bearing situations and ideally reduce the time required for internal/external fixation.

### The role of host and donor in endochondral ossification

Determining the origin of host and donor cells in this type of experiment is a difficult task. Here we used transgenic rats expressing hPLAP into which we implanted scaffolds containing wild type cells of the same inbred strain. This approach has two goals. Firstly, to determine the origin of the bone forming cells in the process of endochondral ossification and secondly to confirm that the results observed in immunocompromised mice could be reproduced in immunocompetent animals. The utility of this approach for the identification of donor/host cells in a variety of tissues has been demonstrated previously [13,14]. In accordance with the experiments using human MSCs in immunocompromised mice, bone formation occurred only under chondrogenic and β-glycerophosphate conditions in rats. Analysis of hPLAP expression in the various tissues clearly demonstrated the presence of host and donor-derived cells. Embedded in the bone matrix, positively and negatively staining cells were observed, suggestive of the presence of cells of both host and donor origin, indicating that at least at earlier time points the donor cells are actively involved in the formation of bone. In order to clearly identify the roles of both host and donor cells, a timecourse analysis coupled with the reverse scenario (Transgenic cells into wild type animals) should be performed. Eight weeks after implantation all osteoblasts and lining cells were of host origin, suggesting that the bone formed from that time point on will be host derived.

## Conclusions

The work presented in this article suggests that the induction of chondrogenesis *in vitro *vs osteogenesis offers an improved approach to bone repair and regeneration *in vivo*. As discussed in a previous article [6], we believe this is in part due to the paracrine effects of these cells with different release profiles of important factors such as VEGF, MMPs and other growth factors at critical stages in the process. It is clear from this work that chondrogenic priming of cells, particularly of adult human MSCs offers an extremely promising route to bone formation and repair that will undoubtedly be pursued in the coming years as an alternative to the standard intramembranous ossification approach of tissue engineering bone.

## Competing interests

The authors declare that they have no competing interests.

## Authors' contributions

EF was involved in study conception and design, cell culture, scaffold seeding in Rotterdam and Vienna and all aspects of analysis and manuscript preparation. SB was involved in study design, performed *in vivo *nude mouse study, analysis of all experimental data and paper preparation. KO performed hPLAP component of study including cell preparation, scaffold seeding and animal surgeries and subsequent hPLAP histochemical analysis. WK was involved in study design, MSC isolation and culture and PCR analysis, NK was involved in all aspects of histological and histochemical analysis, FOB was responsible for scaffold design and fabrication, RBJ was involved in study conception and article preparation, JV was involved in study conception and article preparation, VC performed micro ct analysis and was also involved in histomorphometric analysis, JJ was involved in study conception and article preparation, RE was involved in study conception and design specifically related to hPLAP animal model and article preparation, GvO was involved in study conception and design, data analysis and article preparation. All authors read and approved the final manuscript.

## Pre-publication history

The pre-publication history for this paper can be accessed here:

http://www.biomedcentral.com/1471-2474/12/31/prepub
